# Development of a novel Agarose/Nano-Hydroxyapatite/Grape seed extract hydrogel for biomimetic remineralization of demineralized human enamel (An *In-Vitro* Study)

**DOI:** 10.1038/s41598-025-11490-0

**Published:** 2025-07-18

**Authors:** Hanaa M. Elgamily, Engie M. Safwat, Ahmed M. Youssef

**Affiliations:** 1 Restorative and Dental Materials Department, Oral and dental research institute, National Research Centre, Giza, Egypt; 2 Packaging Materials Department, National Research Centre, 33 El Bohouth St. (former El Tahrir St.), Dokki, P.O. 12622, Giza, Egypt

**Keywords:** Enamel remineralization, Biomimetic regeneration, Agarose hydrogel, Grape seed extract, Hydroxyapatite formation, Non-invasive enamel repair, Environmental sciences, Health care, Nanoscience and technology

## Abstract

Enamel prisms possess a unique microstructure, and their damage due to erosion is irreversible, making enamel restoration through non-invasive regeneration a significant challenge. This *in-vitro* study aimed to reconstruct the prism-like structure of enamel damaged by citric acid erosion through non-invasive biomimetic remineralization. Grape seed extract (GSE), combined with ethylenediaminetetraacetic acid (EDTA) and agarose hydrogel, was prepared via a hydrothermal technique. Additionally, a separate solution containing monoethanolamine (MEA) and potassium phosphate dibasic (K_2_HPO_4_) was prepared. Both solutions were applied as a treatment protocol for 30 h on citric acid-eroded enamel surfaces. Three groups were compared: the control non-eroded enamel group (G0), the eroded non-treated enamel group (G1), and the treated enamel group (G2). The enamel surfaces were analyzed using atomic force microscopy (AFM), scanning electron microscopy coupled with energy dispersive X-ray analysis (SEM-EDX), and transmission electron microscopy (TEM). Significant topographic changes were observed in the G2 group compared to the G0 and G1 groups. AFM analysis revealed the formation of a new layer on the eroded surface as revealed by the increased arithmetical mean deviation of the roughness (Sa = 255.7 ± 40.61 nm) and the smoother surface profile (Sku = 2.98 ± 0.53) of G2 compared to both G0 and G1. SEM examination showed the presence of uniform, prism-like, regenerative tissues, and EDX analysis confirmed the formation of hydroxyapatite (HAp) with a predominant calcium oxide (1.93 Ca/P molar ratio) phase on the treated enamel surface. TEM analysis indicated a crystal size of 12–15 nm. In conclusion, the application of GSE/EDTA agarose hydrogel and MEA/ K_2_HPO_4_ solution successfully repaired the eroded enamel surface, generating a uniform, prism-like enamel structure by providing the necessary inorganic mineral ions and the organic protein matrix template required for biomimetic remineralization.

## Introduction

Enamel, the hardest tissue in the human body, is composed of hierarchically structured, tightly packed prisms, with hydroxyapatite (HAp) crystals arranged parallel within each prism. These HAp crystals have diameters ranging from 25 to 100 nm and lengths between 0.1 and 100 μm. Enamel damage, whether caused by caries, erosion, or other factors, is considered irreversible. Depending on the lesion’s size, restoration is typically achieved through artificial materials or non-invasive remineralization techniques^1,2^. Non-carious enamel lesions, which arise from the loss of hard tissue not caused by caries, can result from prolonged exposure to acids in food and beverages, such as citric acid, phosphoric acid, and carbonic acid. These exposures can lead to enamel erosion and demineralization. Previous treatment protocols for curing enamel erosion, including fluoride varnishes^3^, dentifrices^4^, casein phosphopeptide–amorphous calcium phosphate pastes^5,6^, and bioactive glass-based^7^ and calcium silicate-based restorations^8^ have been explored. While many of these products can remineralize enamel, the resulting crystals are often morphologically irregular with disrupted alignment compared to the structured, prism-like hydroxyapatite of natural enamel^9^.

The pH of therapeutic agents plays a critical role in their interaction with enamel surfaces. An acidic environment promotes demineralization, while a neutral to slightly basic pH favors remineralization by stabilizing calcium and phosphate ion activity. Fluoride, a widely studied cariostatic agent, exerts its protective effect not only by enhancing remineralization but also by reducing enamel solubility in acidic conditions through the formation of fluorapatite. Studies have shown that both ion availability and enamel surface reactivity are pH-dependent^10,11^.

In an attempt to replicate the unique structure of enamel prisms, scientists have explored synthetic approaches involving high temperature, pressure, and acidic pH; however, these methods are impractical for routine dental practice. Consequently, new strategies focusing on biomimetic remineralization of non-carious lesions have been developed^12^.

The development of biomimetic remineralization techniques for treating non-carious lesions requires a comprehensive understanding of dental tissue biomineralization. Biomineralization is the process by which inorganic ions accumulate in a synchronized manner, guided by protein molecules, to form hierarchical structures. Both proteins and inorganic ions play critical roles in driving the systematic biomimetic mineralization process^13^. Three primary theories explain the mechanisms behind biomineralization. The first, the “classical nucleation theory,” suggests that individual ions combine based on kinetic and thermodynamic principles to form ordered clusters, which then evolve into crystal lattices. Protein molecules in this theory help lower the energy barrier, facilitating the formation of properly organized nucleating clusters^14^. The second, the “non-classical nucleation theory,” posits that pre-nucleation clusters aggregate to form well-hydrated, amorphous mineral phases that subsequently crystallize into higher-order structures. These clusters are particularly sensitive to pH and the presence of protein molecules^15,16^. The third, “crystallization by particle attachment,” combines elements of the previous two theories, where mineral phases are created through interactions between ion clusters, gels, and oligomers, leading to the formation of larger crystal assemblies. This process is regulated by energy kinetics, phase stability, and the presence of gels and polymers^17^. Based on these theories, various studies have sought to replicate the biomineralization process using different methodologies^18–21^.

One such approach is the use of metastable amorphous calcium phosphate phases, which support natural enamel remineralization by directly transforming into HAp crystals^22^. Another strategy involves the application of monetite, a calcium phosphate phase known for its ability to resorb quickly in vivo, making it effective in bone regeneration^23^. Monetite, the anhydrous form of brushite, shares similar calcium and phosphorus content with brushite but is more acidic. Both phases have been used as precursors for HAp production and as dental mineralizing agents in products such as chewing gums and toothpastes^24–26^. Given the proven biological adaptability of calcium phosphate phases, numerous studies have investigated novel and cost-effective strategies for synthesizing these phases. In vitro procedures for enamel-like tissue regeneration using various calcium phosphate phases have been well-documented, often involving harsh chemical conditions, such as high-temperature hydrothermal methods^27–29^.

The hydrothermal process is a highly effective and efficient chemical approach for producing various inorganic crystal structures, including HAp in forms such as nanoparticles, nanofibers, nanorods, and plates, as well as monetite crystals^30–33^. In this method, calcium and phosphate salt solutions are typically combined with ammonia or urea to adjust the pH. Organic additives, such as EDTA, are introduced to control the rate of calcium ion release and regulate the solution’s degree of supersaturation. Surfactants are also employed to direct the alignment of nano-apatite rods into a prism-like structure, serving as reverse micro-emulsions. Recently, electrospun nanofibers of amorphous calcium phosphate/poly(vinylpyrrolidone) have been introduced as hydrogel templates to facilitate enamel remineralization in vitro^34–36^. In a related study, agarose hydrogel has been proposed as a biomimetic template for mineralization, mimicking the organic matrix required for mineral deposition in enamel. Agarose is a naturally occurring polysaccharide composed of repeating units of d-galactose and 3,6-anhydro l-galactose^37^. As per the biomineralization theories, the interaction of mineral ions with a gel-like protein matrix is essential for enamel apatite deposition in vivo.

Xie et al.^38^ hypothesized that grape seed extract (GSE) could stabilize the exposed collagen matrix, promoting mineralization by interacting with the organic dentin structure through Proanthocyanidin-collagen binding. Proanthocyanidins (PAs), a class of plant metabolites, have a molecular structure consisting of flavonoids, oligomers, and catechins, and have been shown to support collagen synthesis and prevent ischemia. However, their role in human tooth remineralization remains under investigation^38,39^. Additionally, GSE contains significant amounts of essential minerals such as calcium, potassium, magnesium, sodium, phosphorus, and sulphur, which may aid in mineral precipitation on tooth lesions^38,40^. Monoethanolamine (MEA), an ethanolamine compound with one primary amine group, is an odorless, colorless liquid known for stabilizing solutions and maintaining homogeneity across a wide pH range. A study by Suchanek^41^ demonstrated that MEA could be used to synthesize highly crystalline, pure calcium phosphate structures, including monetite plate-like microcrystals. By adjusting the concentration of MEA, various crystal shapes, including hydroxyapatite plates and nanofibers, could be selectively synthesized.

Despite the promising potential of these materials, many challenges remain in achieving effective enamel regeneration. This study aims to explore an improved biomimetic mineralization strategy for the uniform remineralization of acid-eroded enamel surfaces. We hypothesize that the application of a biomimetic hydrogel containing grape seed extract (GSE) and EDTA, in combination with a monoethanolamine and phosphate solution, can provide protein-guided mineralization models that induce the formation of enamel-like hydroxyapatite crystals on eroded enamel surfaces, thereby mimicking the hierarchical structure of native enamel.

## Materials and methods

### Materials

Red grape seeds (V. vinifera Syrah) were obtained from a grape juice factory (the Gianaclis Grape Juice Factory, Alexandria, Egypt). Citric acid solution (Rasayan Laboratories, was obtained from Anand, Gujarat, India, Batch No. 0396-196-160316). Ethylene diamine tetraacetic acid (EDTA) was obtained from sigma Aldrich, Egypt. Agarose powder (BioWest Regular Agarose G-10, was gotten from Gene Company, Spain). Monoethanolamine (MEA) (Oswal Chemicals, Gujarat, India, CAS No. 75-04-7) and potassium hydrogen phosphate (K_2_HPO_4_) (Thomas-Baker Lab Chemicals, India).

### Preparation of enamel samples and erosive challenge

All procedures were conducted in accordance with the ethical guidelines set forth by the Helsinki Declaration. The experimental protocols were approved by the Medical Research Ethics Committee, National Research Centre of Egypt (**Reference number: 1436072023**). The isolated teeth were obtained from patients undergoing orthodontic extractions at the dental clinic of the Medical and Scientific Centre of Excellence, National Research Centre, Egypt. Informed consent was obtained from all subjects and/or their legal guardians.

Based on power analysis using G*Power software (Faul et al.^42^), a minimum of 25 samples was required to detect a medium effect size (f = 0.25) with 80% power and α = 0.05 in a repeated measures design (within-subject comparison across three conditions). Therefore, 25 sound human premolars were collected and used sequentially in three experimental conditions: G0 (baseline, sound enamel), G1 (after citric acid erosion), and G2 (after biomimetic remineralization). This self-controlled design allowed each tooth to serve as its own control, thereby reducing variability and increasing the study’s sensitivity. Teeth with caries, developmental defects, or surface abnormalities were excluded. Defect-free teeth were ultrasonically cleaned with deionized water and stored in artificial saliva^7^.

For the erosive challenge, the samples were exposed to 50 mL of 0.03 g/10mL citric acid solution (Rasayan Laboratories, Anand, Gujarat, India, Batch No. 0396-196-160316) adjusted to pH 2.77. The samples were incubated for 2 h at 35 °C in a shaking incubator (30 rpm - shaking incubator SI-100R HYSC, Korea)^43,44^.

### Red grape seed extract (GSE) and GSE/EDTA agarose hydrogel preparation

Grape seed extract (GSE) was prepared following the method outlined by Li^45^. The pH-value of the red grape seed extracts was 4.8. Briefly, 200 g of powdered grape seeds were extracted with 800 mL of a 70:30 (v/v) ethanol: water mixture in a shaking incubator at 45 °C for 2 h. The mixture was then centrifuged at 5000 g for 10 min, and the supernatant was decanted. The residue was re-extracted for another 2 h, and the supernatants were collected, evaporated using a rotary evaporator, and stored at −20 °C until further use. A 1.9 mL portion of the prepared GSE was diluted into 100 mL of deionized water. Ethylene diamine tetraacetic acid (EDTA) was added at a 1:0.5 molar ratio, followed by the addition of 0.5 g of agarose powder (BioWest Regular Agarose G-10, Gene Company, Spain). The pH was adjusted to 6.5 using 0.1 M NaOH and 0.1 M HCl. The mixture was allowed to swell at 25 °C for 30 min. For hydrothermal treatment, the mixture was transferred to a 40 mL Teflon-lined stainless-steel reactor, which was placed in a furnace at 100 °C for 12 h. Afterward, the reactor was immersed in an ethylene glycol bath at 0–4 °C for rapid cooling. The hydrogel was then kept at 60 °C until use^36^.

### MEA/K_2_HPO_4_ solution preparation

A solution of monoethanolamine (MEA) (Oswal Chemicals, Gujarat, India, CAS No. 75-04-7) and potassium hydrogen phosphate (K_2_HPO_4_) (Thomas-Baker Lab Chemicals, India) was prepared by adding 2 × 10⁻³ mol/L MEA to a 0.05 mol/L K_2_HPO_4_ solution. The pH of the solution was adjusted to 6.5 using 0.1 M NaOH^46^.

### Biomimetic mineralization model for eroded enamel surfaces using GSE/EDTA agarose hydrogel and MEA/K_2_HPO_4_ solution

For the G2 group (treated enamel), 10 mL of the GSE/EDTA agarose hydrogel was applied to the enamel surface. After allowing the hydrogel to gel for 10 min, then 5 mL of the MEA/K_2_HPO_4_ solution was added, forming a 2-mm-thick layer. Both solutions were maintained on the enamel surface for 30 h at 37 °C in a shaking incubator (30 rpm - shaking incubator SI-100R HYSC, Korea). Following the incubation, the samples were removed, rinsed with deionized water, and air-dried^47^.

### Structural characterization and compositional analysis of the formed crystals forremineralized enamel

#### Surface characterization using AFM

Surface roughness and morphology of the enamel samples before and after acid erosion, as well as after remineralization treatment, were examined using an Atomic Force Microscope (AFM) (Anton Paar - Tosca™ 200, USA). Three-dimensional images of the enamel surfaces were obtained at random sites with a scanning size of 10 μm × 10 μm using an arrow NCR tapping cantilever at 400 × 400 resolution. AFM data were analyzed using Tosca Analysis software according to ISO 25,178 for surface roughness. The roughness parameters evaluated were **Sa**, **Sq**, and **Sku**. **Sa** refers to the arithmetical mean deviation of the roughness, **Sq** is the root mean square deviation of the roughness, and **Sku** refers to the coefficient of kurtosis, which measures the spikiness of the 3D surface texture. A histogram of all measured height points is obtained, with **Sku** representing the deviation from a normal distribution. If **Sku = 3**, the surface exhibits a Gaussian distribution, while **Sku > 3** indicates a spiky surface and **Sku < 3** indicates a bumpy surface^48,49^.

#### SEM-EDX analysis of the remineralized enamel surface

The enamel samples from all three groups were examined using a JEOL JSM-5800 scanning electron microscope under high vacuum conditions, with a chamber pressure maintained at approximately 10⁻⁶ Torr to ensure optimal imaging performance. Sputter coating was not applied in this study because the enamel samples, both before and after treatment, were analyzed under identical conditions, serving as their own control in this self-controlled experimental design. While uncoated biological samples can sometimes lead to charging effects, the enamel surfaces provided sufficient conductivity for imaging due to their inherent mineral content and smoothness. An Energy Dispersive X-ray Spectrometer (EDX) with a SiLi X-ray detector (Oxford Instruments, UK) was employed for elemental analysis of the enamel surfaces^33^.

#### TEM analysis for identification of the obtained crystals on the remineralized enamel surface

The tooth samples were fixed using a mixture of 2.5% glutaraldehyde and 1.5% paraformaldehyde in 0.1 M cacodylate buffer (pH 7.4) for 30 min. After washing three times with 0.1 M cacodylate buffer, post-fixation was carried out using osmium tetroxide for 1 h. The samples were dehydrated in an ascending ethanol series and post-stained with uranyl acetate. Enamel samples were sectioned into 3 × 3 mm slices with a thickness of 250–500 μm using a diamond disc. These were cleaned in a solution of isopropanol and acetone (60%:40%, v/v) for 15 min^50^. The transmission electron microscope (TEM) (JEOL JEM-210) system, equipped with a field emission.

### Statistical analysis

Data were statistically analyzed using Microsoft Excel^®^ 2016, Statistical Package for Social Sciences (SPSS^®^) Version 24, and Minitab^®^ Version 16. One-way Analysis of Variance (ANOVA) was performed to compare the outcomes (remineralization effects on enamel) across the different groups. Post-hoc tests were conducted when appropriate to identify specific group differences. Data from three replicates are presented as mean ± standard deviation (SD).

## Results and discussion

### AFM analysis

**Fig. 1:**
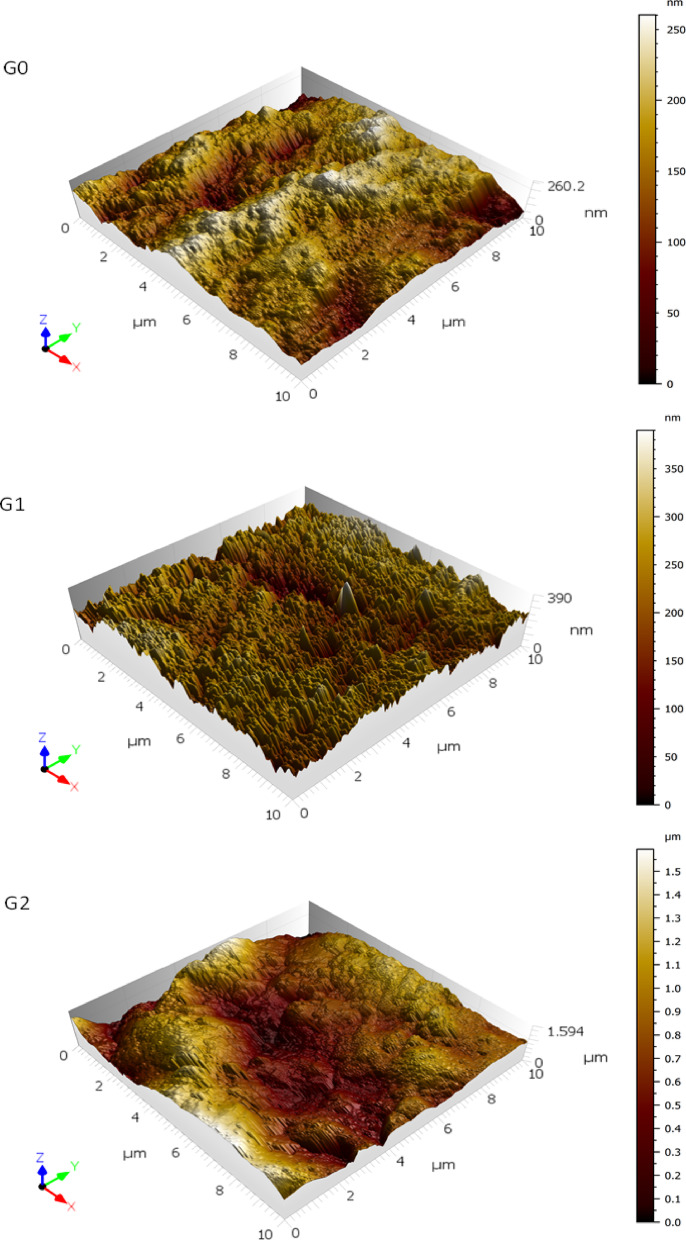
3D AFM images of the non-eroded enamel surface **(G0)**, eroded enamel surface **(G1)** and treated enamel surface **(G2)**.

AFM analysis revealed significant alterations in the topography of the enamel surface after citric acid erosion and subsequent remineralization treatment (Fig. 1 and Table 1). The enamel surface, following citric acid erosion, appeared highly rugged, with increased surface roughness. However, after remineralization treatment, this ruggedness was mitigated, and the enamel surface became even smoother than its baseline condition before acid erosion. Specifically, the mean roughness parameters (Sq and Sa) increased from 96.31 ± 54.79 nm and 77.26 ± 44.78 nm in the G0 (control) group to 170.48 ± 128.1 nm and 111.03 ± 79.28 nm in the G1 (acid-eroded) group, respectively. Upon remineralization (G2 group), a new layer was deposited, significantly elevating the enamel surface, with higher Sq and Sa values of 318.05 ± 52.45 nm and 255.7 ± 40.61 nm, respectively.

**Table 1:** Mean roughness results parameters of non-eroded samples (G0), eroded samples (G1) and eroded samples (G2) in nm.

Roughness parameters	G0 mean (nm)	G1 mean (nm)	G2 mean (nm)
**Sq**	96.31 ± 54.79	170.48 ± 128.1	318.05 ± 52.45
**Sa**	77.26 ± 44.78	111.03 ± 79.28	255.7 ± 40.61
**Sku**	3.01 ± 0.47	5.99 ± 1.59	2.98 ± 0.53

Regarding roughness profile sharpness, as measured by kurtosis (Sku), the value for the G0 group was 3.01 ± 0.4, indicating a normal distribution of surface heights. However, this value increased to 5.99 ± 1.5 for the G1 group, suggesting that the height distribution became more spiked due to acid erosion. After remineralization treatment in the G2 group, Sku values decreased to 2.98 ± 0.53, indicating a smoother surface profile. This shift from sharp spikes to round bumps reflects the deposition of a new layer, which helped restore the enamel surface topography. The citric acid erosion significantly altered the surface of the enamel, as evidenced by the increased roughness values in G1 group, caused by the dissolution of HAp crystals within the enamel prisms. After remineralization treatment in the G2 group, a new mineralized layer was deposited, which elevated the surface height, as seen in the increased Sq and Sa values.

These results reveal the impact of biomimetic remineralization on enamel surface and suggest that the remineralization treatment effectively restored the enamel surface to a condition similar to its baseline before erosion. The kurtosis (Sku) values provided additional insight into the surface profile. While the G1 group exhibited a highly spiked surface profile after acid erosion, the G2 group showed a smoother, more rounded surface profile following remineralization. This indicates that the remineralization process not only restored mineral content but also improved the overall surface morphology of the enamel.

### SEM-EDX analysis

**Fig. 2:**
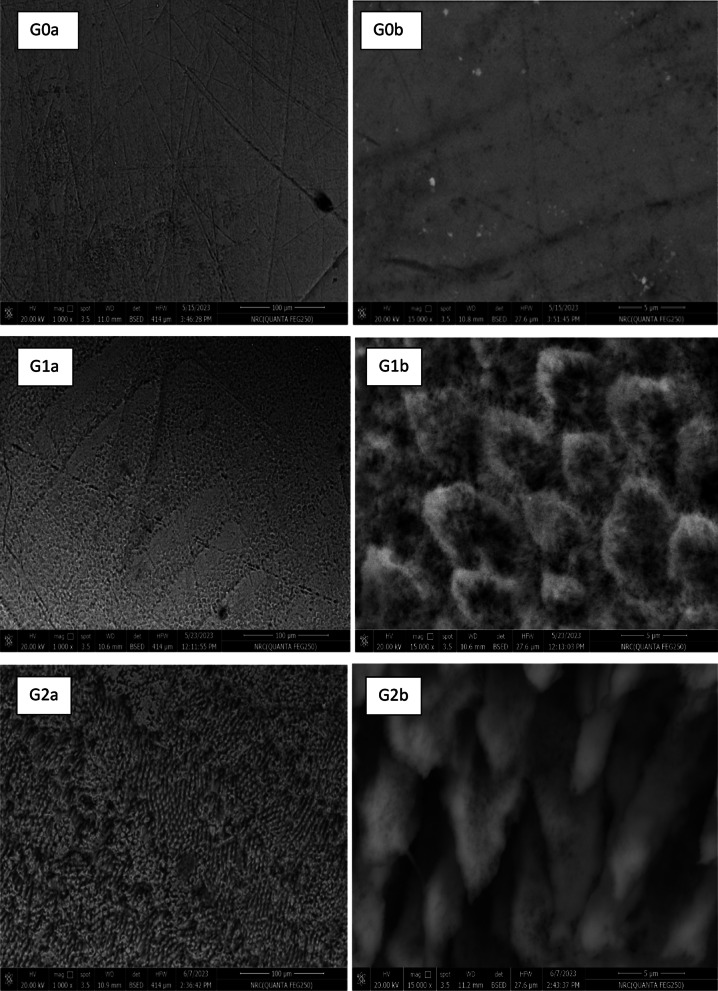
SEM images of teeth surfaces of the non-eroded **(G0)**, eroded **(G1)**, and treated **(G2)** enamel surface at magnification of 1000× **(a)** and 15,000× **(b)**.

SEM images magnified at 1000× and 15,000× of the non-eroded (G0), eroded (G1), and remineralized (G2) enamel surfaces are shown in (Fig. 2). The G0 group exhibited a relatively smooth surface with intact enamel prism cores. After citric acid erosion (G1 group), then the enamel surface showed dissolution of the prism cores, leaving the prism periphery largely intact. In the G2 group, remineralization treatment led to the closure of open prism cores by uniformly distributed insoluble crystalline precipitates, which were formed within the prism cores and across the enamel surface. These SEM images explain the previously obtained AFM results where G1 revealed increased roughness parameters (Sa and Sq) as a result of the resolved prism core that increased the depth of the enamel surfaces, however, G2 showed increase in the surface height due to the new deposited minerals inside the prism core.

**Fig. 3:**
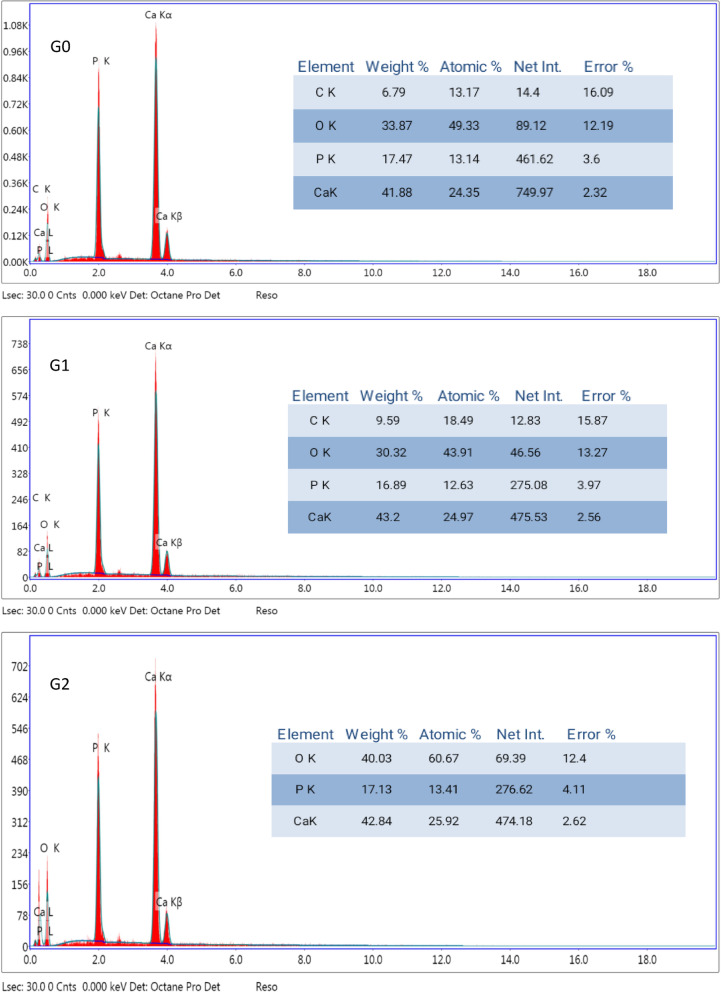
EDX Spectra analysis of the non-eroded enamel surface **(G0)**, eroded enamel surface **(G1)** and treated enamel surface **(G2)**.

Energy-dispersive X-ray (EDX) analysis showed no significant differences in the calcium and phosphorus content (weight%) between the groups (Fig. 3). The calculated Ca/P molar ratios were 1.85, 1.97, and 1.93 for the G0, G1, and G2 groups, respectively, suggesting a similar mineral composition across all groups. These findings are consistent with previous studies that reported comparable Ca/P ratios in enamel-like materials^51,52^. The lack of significant differences in the Ca and P weight percentages across the groups, as observed in EDX analysis, may be due to the presence of residual Ca^2+^ ions on the surface of the eroded enamel (G1 group) and the similarity in Ca/P content between the remineralized (G2) and control (G0) groups. The slight differences in the Ca/P molar ratios (1.85 for G0, 1.97 for G1, and 1.93 for G2) are in line with the typical Ca/P ratio of enamel, which is approximately 2.13 ± 0.17^52^. A previous study^53^ examined samples prepared with intentionally adjusted Ca/P ratios ranging from 0.5 to 2.5. The study found that samples with a 2.0 Ca/P ratio predominantly presented the HAp phase, with the detection of CaO formation.

### TEM analysis

**Fig. 4:**
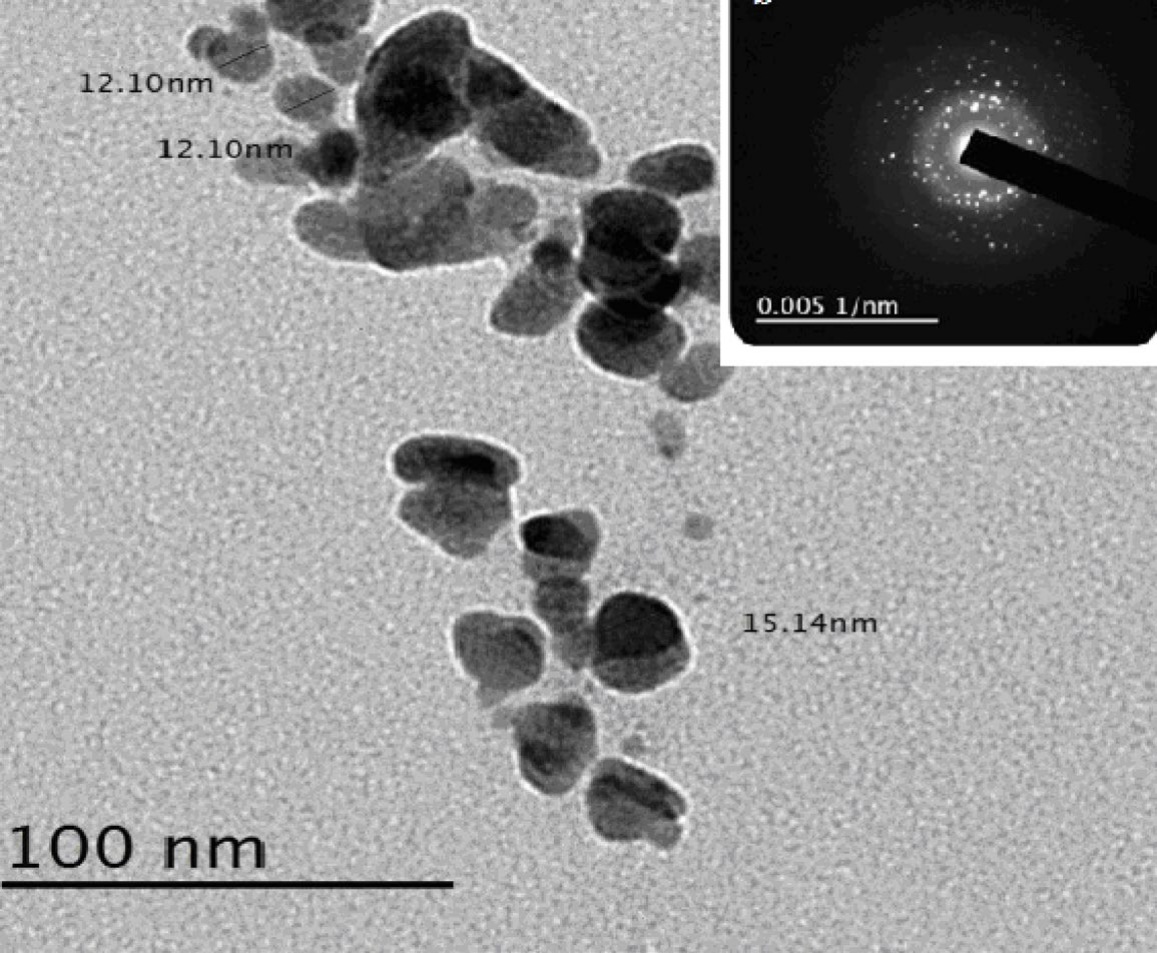
TEM image and SEAD pattern of HAp crystals synthesized by MEA - GSE/EDTA agarose hydrogel.

TEM images (Fig. 4) of the remineralized enamel surfaces revealed the formation of hydroxyapatite (HAp) crystals, which were approximately 12–15 nm in size and aggregated into clusters. Selected-area electron diffraction (SAED) patterns indicated the crystalline nature of the deposited minerals, which corresponds with the SEM images (Fig. 2). The TEM analysis provides essential insight into the morphology and crystallinity of the mineral phase generated by the hydrogel, thereby supporting the proposed biomimetic mechanism of enamel remineralization. Including this figure establishes the hydrogel’s intrinsic capacity to nucleate and grow enamel-like apatite, which is critical for interpreting its remineralizing effect observed in the treated enamel samples.

The presence of a dark region in the central area of the crystals could suggest a transition from octacalcium phosphate (OCP) to hydroxyapatite (HAp), as both phases exhibit mismatched layers^54^. However, the more plausible explanation is that this dark area represents a Ca-rich region within the crystals, which aligns with known observations that enamel crystals begin to dissolve at their central regions during acid exposure^55^. Therefore, in the present study, the erosive citric acid attack began centrally, after which the remineralization process, facilitated by the GSE/EDTA agarose hydrogel and MEA/K_2_HPO_4_ solution, promoted the deposition of minerals over these lesions, thereby restoring the enamel’s integrity. In addition, previous studies^56,57^ have stated that calcium atoms in the HAp unit cell reveal a higher defect density and atomic overlap, as human enamel crystals are susceptible to electron beam radiation damage during TEM observation. As a result, the TEM electron beam amplified the overlap of HAp atoms during crystal examination^58^. Since the HAp crystals had nanometric dimensions, as revealed in the electron diffraction pattern, the incident electron beam’s minimum cross-section was beyond 10 nm, meaning the crystals’ nanometric region, from which the electron diffraction patterns originated, essentially encompassed the entire human tooth enamel crystal.

### Implications of the remineralization approach

While the current study provides strong morphological evidence for enamel remineralization using AFM, SEM, and TEM imaging, it is important to acknowledge that mineral content was not quantified using transverse microradiography (TMR), additionally, substance loss during the erosion-remineralization cycle was not directly measured. These aspects represent important limitations, as TMR could have offered more accurate insight into lesion depth changes and true mineral gain. Future studies incorporating TMR and profilometry or optical coherence tomography (OCT) are needed to quantitatively assess mineral uptake and enamel loss.

However, the surface and subsurface changes observed via AFM, SEM-EDX, and TEM in this study align with previous reports indicating that surface morphology improvements often correlate with remineralization efficacy, even in the absence of TMR. For instance, a previous study^59^ demonstrated that gum containing calcium fluoride significantly reinforced enamel subsurface lesions in-situ by promoting mineral diffusion, despite using microradiography as a semi-quantitative measure. Their findings underscore the relevance of morphological improvements in supporting remineralization potential, particularly when accompanied by high-affinity mineral-binding agents like proanthocyanidins in grape seed extract (GSE).

Furthermore, the remineralization of enamel via the biomimetic approach using GSE/EDTA agarose hydrogel and MEA/K_2_HPO_4_ solution has shown promising results in restoring enamel integrity. This process mimics the natural enamel biomineralization process and is particularly effective in sealing the eroded enamel prisms, as observed through both AFM and SEM analyses. The incorporation of GSE, rich in proanthocyanidins, further enhances the remineralization process by facilitating calcium ion deposition and strengthening the organic matrix of the enamel^38,60,61^.

The pH of the remineralizing environment is a critical determinant of the efficacy of enamel repair. In this study, the use of monoethanolamine (MEA) served not only as a pH buffer but also contributed to stabilizing the ionic environment favorable for hydroxyapatite (HAp) formation. A previous study^41^ found that, MEA allows the stabilization of the initial solution across a wide pH range and therefore expands the ability of the synthesis method to a broader selection of calcium phosphate phases.

A slightly basic pH, as maintained by MEA, promotes the supersaturation of calcium and phosphate ions, thereby favoring mineral precipitation over demineralization. This aligns with fluoride-based research, where pH modulation was shown to significantly influence mineral uptake. Kitasako et al.^59^, demonstrated that chewing gum containing calcium fluoride enhanced enamel subsurface remineralization and emphasized the importance of maintaining an optimal pH to support ion diffusion and crystal formation. Similarly, in the present study, the controlled alkaline conditions supported by MEA facilitated the nucleation of enamel-like HAp nanocrystals, as observed under TEM. This highlights the synergistic role of pH not just as a passive medium but as an active modulator of mineralization kinetics and phase stability.

However, the roles of acidic and alkaline pH in fluoride’s dental interactions are distinct and complementary, representing two sides of the demineralization-remineralization cycle. Under highly acidic conditions, as demonstrated by Scholz et al.^62^, a primary interaction is the formation of a thick calcium fluoride (CaF_2_) precipitate on the enamel. This process is critically dependent on the acid itself, which first dissolves the enamel surface to release the necessary calcium ions that then react with fluoride to form this protective layer. In contrast, an alkaline or neutral pH is the essential driving force for remineralization, as highlighted by Kitasako et al.^59^, where stimulated saliva raises the oral pH to this alkaline state, creating an environment supersaturated with calcium and phosphate ions that are driven back into the tooth to repair subsurface lesions. These two pH-dependent mechanisms are linked: the CaF_2_ layer formed during an acid attack can later act as a pH-controlled reservoir, releasing fluoride ions to strengthen and enhance the natural remineralization process that occurs under favorable alkaline conditions.

As a result, the remineralization strategy presented in this study effectively regenerated enamel surfaces that had been eroded by citric acid. The deposition of a new mineralized layer, the restoration of surface roughness, and the sealing of enamel prism cores all point to the potential of this biomimetic approach in preventive dental care and enamel regeneration. Our hypothesis suggests that the GSE/EDTA agarose hydrogel and MEA/K_2_HPO_4_ solution act synergistically to promote mineral deposition on the acid-eroded enamel, facilitating the formation of a new, structured mineralized layer that mimics the natural enamel surface. The results support the notion that this remineralization strategy not only restores the mechanical and topographical properties of the enamel but also provides a promising method for enhancing the resilience of enamel against further erosive attacks.

## Conclusion

The biomimetic synthesis process presented in this study, utilizing GSE/EDTA agarose hydrogel and MEA/K_2_HPO_4_ solution, offers a novel approach for generating prism-like enamel structures through the complex nucleation and uniform growth of inorganic crystal formations, guided by organic protein matrix templates. While further in vivo studies are needed to evaluate the efficacy of this remineralization treatment in situ, this research represents a significant step toward the development of innovative biomaterials for reparative and restorative dentistry, with the potential to regenerate enamel-like structures.

## Data Availability

The datasets used and/or analysed during the current study are available from the corresponding author on reasonable request.

## Abbreviations

GSE: Grape seed extract
EDTA: ethylene diamine tetra acetic acid
MEA: monoethanolamine
K_2_HPO_4_: potassium phosphate dibasic
AFM: atomic force microscope
SEM-EDX: scanning electron microscopy coupled with energy dispersive X-ray analysis
TEM: transmission electron microscopy
Hap: hydroxyapatite
PA: Proanthocyanidin
